# Inoculum selection influences the biochemical methane potential of agro-industrial substrates

**DOI:** 10.1111/1751-7915.12268

**Published:** 2015-03-10

**Authors:** Jo De Vrieze, Linde Raport, Bernard Willems, Silke Verbrugge, Eveline Volcke, Erik Meers, Largus T Angenent, Nico Boon

**Affiliations:** 1 Laboratory of Microbial Ecology and Technology (LabMET), Ghent UniversityCoupure Links 653, Gent, B-9000, Belgium; 2 Department of Biosystems Engineering, Ghent UniversityCoupure Links 653, Gent, B-9000, Belgium; 3 Laboratory of Analytical Chemistry and Applied Biochemistry, Ghent UniversityCoupure Links 653, Gent, B-9000, Belgium; 4 InnolabDerbystraat 223, Sint-Denijs-Westrem, 9051, Belgium; 5 Department of Biological and Environmental Engineering, Cornell UniversityIthaca, NY, 14853, USA

## Abstract

Obtaining a reliable estimation of the methane potential of organic waste streams in anaerobic digestion, for which a biochemical methane potential (BMP) test is often used, is of high importance. Standardization of this BMP test is required to ensure inter-laboratory repeatability and accuracy of the BMP results. Therefore, guidelines were set out; yet, these do not provide sufficient information concerning origin of and the microbial community in the test inoculum. Here, the specific contribution of the methanogenic community on the BMP test results was evaluated. The biomethane potential of four different substrates (molasses, bio-refinery waste, liquid manure and high-rate activated sludge) was determined by means of four different inocula from full-scale anaerobic digestion plants. A significant effect of the selected inoculum on the BMP result was observed for two out of four substrates. This inoculum effect could be attributed to the abundance of methanogens and a potential inhibiting effect in the inoculum itself, demonstrating the importance of inoculum selection for BMP testing. We recommend the application of granular sludge as an inoculum, because of its higher methanogenic abundance and activity, and protection from bulk solutions, compared with other inocula.

## Introduction

The remarkable growth of the biogas industry in the world in recent years has launched an intensive search for new organic waste streams to serve as substrates for anaerobic digestion (Appels *et al*., 2011). Substrates for anaerobic digestion can be characterized by means of a biochemical methane potential (BMP) test that allows the determination of the biodegradability and the associated methane production potential during anaerobic digestion of a given substrate (Owen *et al*., 1979). The BMP of a biomass substrate is an important factor for its price setting, when being purchased by biogas plant owners, and is therefore considered a key parameter to estimate the profit margins of the anaerobic digestion plant. Several studies were carried out over the last few years, demonstrating an increasing interest in methods for accurate measurement of the BMP of these biomass substrates (Lesteur *et al*., 2010; Labatut *et al*., 2011; Raposo *et al*., 2011; 2012).

The anaerobic digestion process involves biological conversions in which a consortium of interdependent microorganisms is responsible for the degradation of complex organic matter (Angenent *et al*., 2004). Therefore, an estimation of methane production based on the chemical composition of the substrate is not sufficient, despite the availability of well-developed complex models, and a BMP measurement is preferred (Angelidaki *et al*., 1999; Labatut *et al*., 2011). However, standardization of these BMP tests is complex, but necessary to obtain reliable, reproducible and universal results. Guidelines for the determination of the BMP in batch assays have been proposed by Angelidaki and colleagues (2009), including the substrate characterization, the inoculum and its activity, the experimental procedure and the collection, interpretation and reporting of the data. They suggested for the inoculum to be ‘fresh’, homogenous, sieved and pre-incubated, to have a wide microbial diversity to ensure a sufficient level of hydrolytic and methanogenic activity, and to be tested towards model substrates, such as cellulose and acetic acid (Angelidaki *et al*., 2009). However, no recommendations about the methanogenic community composition or abundance were included. Raposo and colleagues (2012) reviewed the factors affecting the performance of anaerobic batch assays, and indicated that, although experimental conditions of batch assays are synchronized, a certain degree of variability in the results always remains due to the biological nature of the test systems. This biological difference can be assigned to the origin of the inoculum, as it comes with a different microbial population, leading to differences in initial activity and substrate adaptation (Wittebolle *et al*., 2009; Regueiro *et al*., 2012; Gough *et al*., 2013). Therefore, different inocula will show a difference in the ability to convert the substrate to methane. The influence of the inoculum source on methane production has recently been indicated by Chamy and Ramos (2011) for turkey manure and by Elbeshbishy and colleagues (2012) for food waste and primary sludge, and in both cases the methane yield depended on the inoculum sample that was selected. However, this influence was not linked to the characteristics of the microbial community in the different inocula.

The aim of the present study was to evaluate the specific contribution of the methanogenic community on the results of BMP testing. For this purpose, the BMP of each of the four different substrates was evaluated by means of four different inocula. We selected an inoculum in terms of its ‘fitness’ to be used as standard inoculum for BMP batch assays, through characterization of its methanogenic community.

## Results

### Inoculum characterization

Anaerobic sludge samples from four different full-scale mesophilic anaerobic digestion plants were selected to determine the BMP of four different substrates, as to evaluate the effect of inoculum selection on the BMP results. Due to the difference in substrate and reactor configuration of the full-scale facilities, substantial differences in inocula characteristics were detected (Table 1). The brewery wastewater (BREW) inoculum originated from an upflow anaerobic sludge blanket (UASB) digester treating brewery wastewater, and consisted of granular sludge, unlike the other inocula. The granules were not disintegrated prior to the BMP tests to avoid disturbance of the microbial community in the granules.

**Table 1:** Characteristics of the four different inocula sludge samples (*n* = 3)

Parameter	Unit	OBW	MAN	BREW	ENG
pH	–	8.10 ± 0.00	8.28 ± 0.02	6.85 ± 0.01	7.34 ± 0.01
TS	g TS l^−1^	159.9 ± 1.3	121.6 ± 1.8	51.5 ± 1.7	88.6 ± 1.7
VS	g VS l^−1^	86.4 ± 1.4	79.0 ± 1.8	42.8 ± 1.5	66.4 ± 1.8
Conductivity	mS cm^−1^	33.9 ± 1.6	39.3 ± 0.2	4.8 ± 0.0	14.9 ± 0.4
Total VFA	mg COD l^−1^	0 ± 0	72 ± 0	0 ± 0	1397 ± 338
Acetic acid	mg COD l^−1^	0 ± 0	0 ± 0	0 ± 0	542 ± 109
Propionic acid	mg COD l^−1^	0 ± 0	72 ± 0	0 ± 0	216 ± 52
TAN	mg N l^−1^	4363 ± 1170	5144 ± 820	100 ± 21	1289 ± 257
FA[Table-fn tf1-1]	mg N l^−1^	514 ± 138	859 ± 137	1 ± 0	29 ± 6

The free ammonia (FA) content was calculated based on the TAN concentration, pH and temperature in the full-scale installation (Anthonisen *et al*., 1976).

The organic biological waste (OBW) and mainly animal manure (MAN) inocula showed higher pH, conductivity and total ammonia nitrogen total ammonia nitrogen (TAN) values, compared with the BREW and energy crops and manure (ENG) inocula (Table 1), which can be attributed to the digestion of high amounts of manure in both plants, which was not the case in the ENG plant. Low residual volatile fatty acids (VFA) concentrations were present in the four inocula, although a total VFA concentration of 1397 mg chemical oxygen demand (COD) l^−1^ was measured in the ENG inoculum (Table 1).

### Substrate characterization

Four different substrates (i.e. molasses, bio-refinery waste, liquid manure and A-sludge) were selected, because of their different characteristics and their (future) importance for renewable energy production by means of anaerobic digestion (Table 2). Both molasses and the bio-refinery waste are characterized by a high total COD content (Table 2), and, therefore, can be considered highly suitable substrates for anaerobic digestion. Molasses, in contrast to bio-refinery waste has a high total Kjeldahl nitrogen (TKN) and salt content. Also, the pH of the molasses was almost two units higher than the bio-refinery waste. Liquid manure is a waste stream often treated by means of anaerobic digestion. The liquid manure, however, has a much lower COD content and higher TAN content, making it a less suitable substrate for anaerobic digestion. A-sludge is harvested from a high-rate activated sludge system, or A-stage, of the ‘Adsorptions-Belebungsverfahren’ or A/B process for municipal wastewater treatment (Boehnke *et al*., 1997). This A-stage allows conversion of the organic matter in the wastewater to microbial biomass at lower sludge retention time values of 2–3 days compared with conventional activated sludge systems (Boehnke *et al*., 1997; Ge *et al*., 2013). The COD content of the A-sludge is higher compared with liquid manure, but TAN and TKN concentrations are lower.

**Table 2:** Characteristics of the four different substrates

Parameter	Unit	Molasses	Bio-refinery waste	Liquid manure	A-sludge
pH	–	5.68 ± 0.01	3.79 ± 0.01	7.89 ± 0.01	6.40 ± 0.03
TS	g TS kg^−1^ FW	504.6 ± 0.0	144.2 ± 0.4	20.5 ± 0.1	23.9 ± 0.1
VS	g VS kg^−1^ FW	342.1 ± 2.5	135.9 ± 0.3	11.4 ± 0.2	19.0 ± 0.1
Total COD	g COD kg^−1^ FW	445.2 ± 6.8	249.7 ± 9.8	21.6 ± 0.7	32.1 ± 1.2
Soluble COD	g COD kg^−1^ FW	405.7 ± 5.2	20.3 ± 0.5	4.7 ± 0.1	1.7 ± 0.0
Conductivity	mS cm^−1^	25.7 ± 0.1	7.6 ± 0.1	29.4 ± 0.1	2.2 ± 0.0
Total VFA	mg COD kg^−1^ FW	5808 ± 256	3693 ± 72	119 ± 4	1248 ± 68
Acetic acid	mg COD kg^−1^ FW	5670 ± 250	3693 ± 72	119 ± 4	314 ± 27
Propionic acid	mg COD kg^−1^ FW	139 ± 6	0 ± 0	0 ± 0	272 ± 18
TAN	mg N kg^−1^ FW	1572 ± 130	568 ± 23	2640 ± 288	235 ± 86
TKN	mg N kg^−1^ FW	20 783 ± 5199	3781 ± 544	2681 ± 160	1458 ± 424
Total P	mg P kg^−1^ FW	4870	812	283	513
COD: N ratio	–	21.4 ± 5.4	66.0 ± 9.9	8.1 ± 0.5	22.0 ± 6.5
COD: P ratio	–	91.4 ± 1.4	307.6 ± 12.1	76.5 ± 2.3	62.6 ± 2.3
TS : VS ratio	–	1.48 ± 0.01	1.06 ± 0.00	1.79 ± 0.03	1.26 ± 0.01
COD : VS ratio	–	1.30 ± 0.02	1.84 ± 0.07	1.90 ± 0.07	1.69 ± 0.06

All analyses are carried out in triplicate, with the exception of the total P analysis. FW = fresh weight.

### Inoculum effect on the BMP results of the different substrates

**Figure 1:**
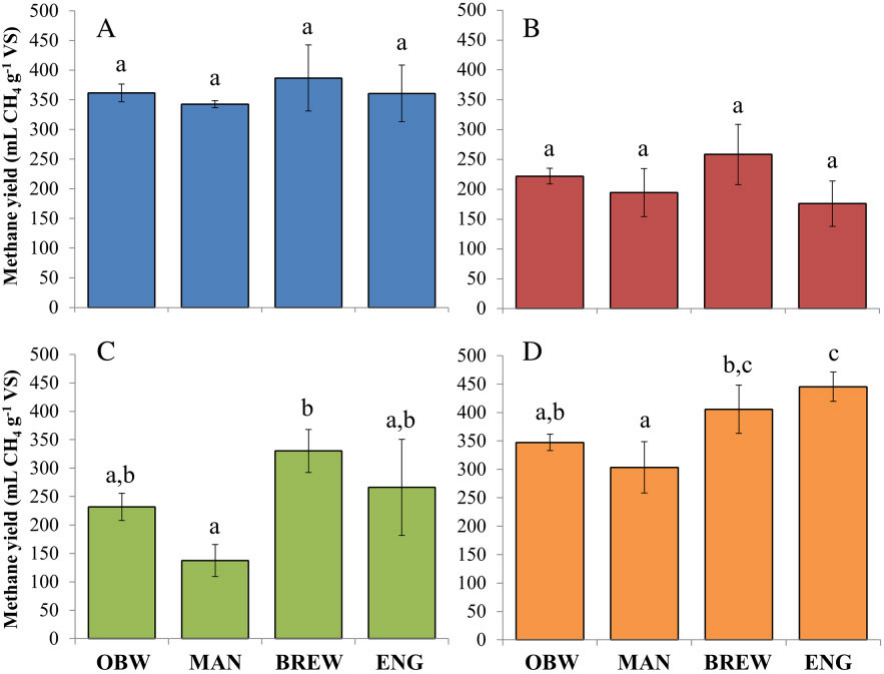
Ultimate methane yields (ml CH_4_ g^−1^ VS) of the (A) molasses, (B) bio-refinery waste, (C) liquid manure, and (D) A-sludge substrates. Error bars show standard deviations, and different letters (A, B and C) indicate significant differences according to ANOVA and subsequent multiple comparison by a Tukey hsd test at the 5% significance level.

Incubation with different inocula resulted in similar BMP values for molasses, because no significant differences were observed between the different inocula (Fig. 1A). An average value of 363 ± 18 ml CH_4_ g^−1^ volatile solids (VS) was observed, which related to a COD conversion efficiency of 79.7 ± 4.0%. Similar to molasses, no significant differences were observed between the different inocula for the BMP determination of the bio-refinery waste substrate (Fig. 1B). An average methane yield value of 213 ± 36 ml CH_4_ g^−1^ VS and COD conversion efficiency of 33.1 ± 5.6% were obtained. The BMP test with liquid pig manure as substrate showed a significant difference in BMP results between the MAN and BREW inoculum (Fig. 1C), with a value of 137 ± 28 and 330 ± 38 ml CH_4_ g^−1^ VS, and a COD conversion efficiency of 20.7 ± 4.3 and 49.7 ± 5.7% for the MAN and BREW inocula respectively. The determination of the BMP of A-sludge resulted in a significant higher BMP value for the ENG inoculum (Fig. 1D), compared with the OBW and MAN inocula, with methane yield values of 455 ± 26, 347 ± 14 and 304 ± 45 ml CH_4_ g^−1^ VS, and COD conversion efficiencies of 75.2 ± 4.3, 58.6 ± 2.4 and 51.2 ± 7.6% for the ENG, OBW and MAN inocula respectively. The BREW inoculum (methane yield of 406 ml CH_4_ g^−1^ VS and COD conversion efficiency of 68.5%) also reached a significant higher BMP result than the MAN inoculum for the A-sludge.

**Figure 2:**
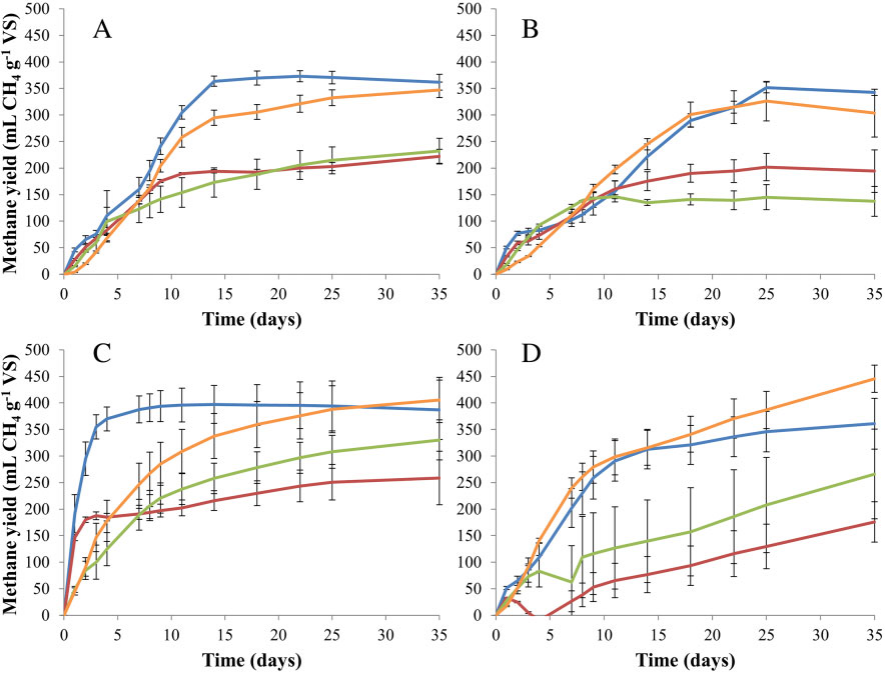
Methane yield curves of the molasses (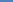), bio-refinery waste (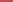), liquid manure (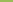), and A-sludge (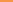) substrates for the (A) OBW, (B) MAN, (C) BREW and (D) ENG inocula. Error bars show standard deviations.

The OBW and MAN inocula showed similar final methane yield results for all four substrates, although methane yield was always slightly higher for the OBW inoculum (Fig. 1A and B). In accordance, a similar methane production profile in function of time was observed for both inocula (Fig. 2A and B). The BREW inoculum, with the exception of the A-sludge substrate, produced the highest methane yield values for each substrate. Moreover, methane production showed very high values, especially for the molasses and bio-refinery waste substrates, during the first few days of the BMP experiment, indicating high methanogenic activity (Fig. 2C). These three inocula (OBW, MAN and BREW) reached a clear plateau phase after 35 days. The ENG inoculum showed a difficult start-up for each sample, indicating low methanogenic activity, and the plateau phase, apparently, was not yet obtained after 35 days (Fig. 2D). The methane yield of the negative control treatments is presented in the Supporting Information (SI) (Fig. S1).

**Figure 3:**
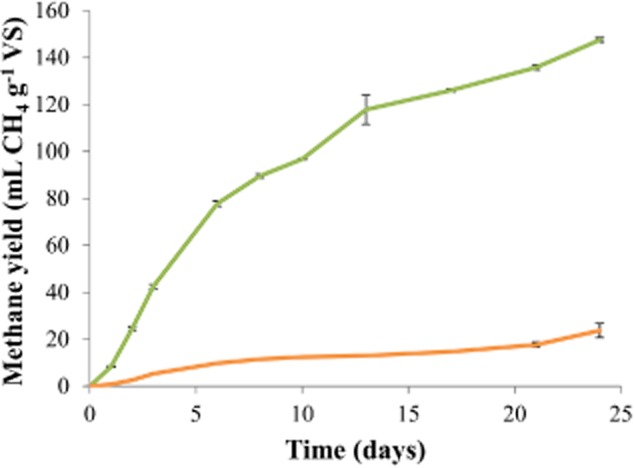
Methane yield curves of the liquid manure (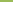), and A-sludge (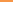) substrates incubated without the addition of an inoculum. The molasses and bio-refinery waste substrates were not included in the figure, because of these substrates did not show any indigenous methanogenic activity. Error bars show standard deviations.

Incubation of the different substrates (without inoculum) to estimate their indigenous methanogenic activity clearly showed that only liquid manure and A-sludge possessed indigenous methanogenic activity, as methane yield values of 148 ± 1 and 24 ± 3 ml CH_4_ g^−1^ VS were obtained for the liquid manure and A-sludge respectively (Fig. 3). However, these methane yield values for the substrates in the absence of an inoculum were much lower than the BMP tests in which an inoculum was used. No methane production was observed during the incubation of the molasses and bio-refinery waste without inoculum.

### Microbial community of the inoculum and substrates

**Figure 4:**
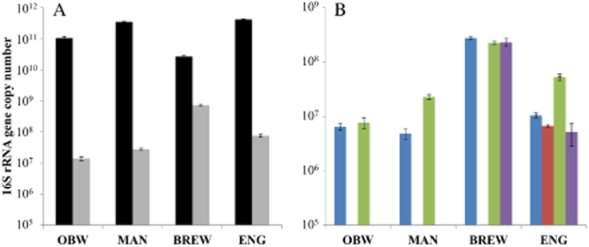
Real-time PCR results of the microbial community in the different inocula, showing (A) total bacteria (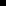) and total methanogens (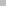) and (B) the methanogenic populations Methanosaetaceae (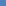), Methanosarcinaceae (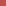), Methanobacteriales (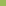), and Methanomicrobiales (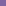). Error bars show standard deviations.

Total bacteria copy numbers were similar in the three inocula OBW, MAN and ENG. Total bacteria abundance was roughly a factor 10 lower and total methanogens abundance 10 times higher in the BREW inoculum compared with the other inocula (Fig. 4A). A similar methanogenic population was observed (only Methanosaetaceae and Methanobacteriales were detected) in the OBW and MAN inocula (Fig. 4B). A different methanogenic community profile was observed in the BREW and ENG inocula. The BREW inoculum hosted an equally high abundance of Methanosaetaceae, Methanobacteriales and Methanomicrobiales, while all four methanogenic groups were represented in the ENG inoculum, with the Methanobacteriales population being the most abundant (Fig. 4B). In all four inocula, Methanosaetaceae and Methanobacteriales were detected, indicating that methane production took place by means of both the acetoclastic and hydrogenotrophic pathway in all full-scale plants from which the inocula originated.

All substrates contained high levels of total bacteria, i.e. between 3.5 × 10^9^ and 3.6 × 10^11^ copies g^−1^. The liquid manure substrate contained Methanosaetaceae, Methanobacteriales and Methanomicrobiales at copy number values of 2.8 × 10^8^ ± 8.6 × 10^6^, 4.1 × 10^8^ ± 2.0 × 10^7^ and 3.5 × 10^7^ ± 1.4 × 10^7^ copies g^−1^, respectively, whereas the A-sludge substrate contained only Methanosaetaceae (2.1 × 10^7^ ± 1.8 × 10^6^ copies g^−1^) and Methanobacteriales (1.3 × 10^8^ ± 1.4 × 10^7^ copies g^−1^). No methanogens were detected in the molasses and bio-refinery substrates.

### Methanogenic community evolution

**Figure 5:**
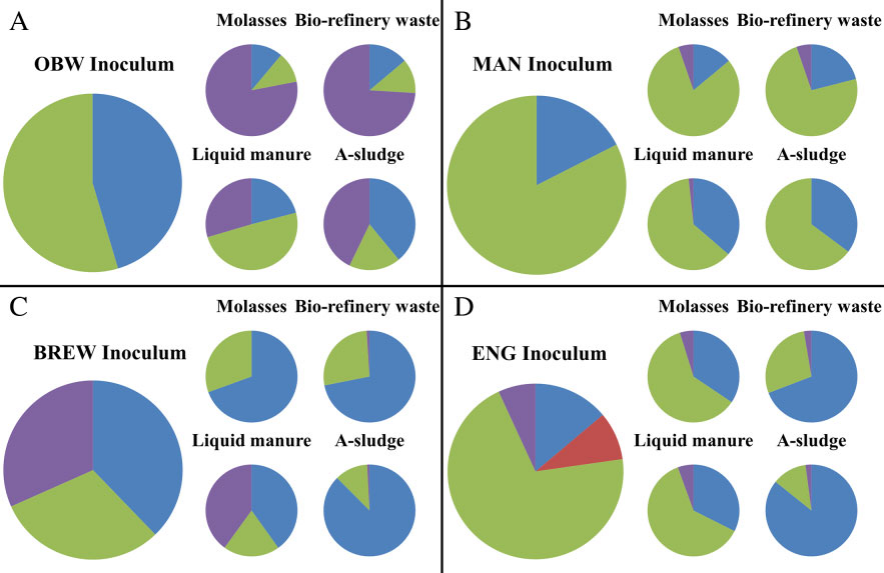
Real-time PCR results of the methanogenic community, showing the Methanosaetaceae (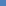), Methanosarcinaceae (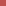), Methanobacteriales (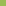), and Methanomicrobiales (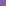). Relative abundances are presented at the beginning (initial inoculum, big graphs) and at the end of the BMP test (after 35 days, small graphs) for the (A) OBW, (B) MAN, (C) BREW, and (D) ENG inocula for the different substrates.

Application of the OBW inoculum resulted in a strong increase in relative abundance of the Methanomicrobiales at the end of the BMP test, irrespective of the substrate, and despite the fact that Methanomicrobiales abundance was below detection limit in the initial inoculum (Fig. 5A). The methanogenic community in the MAN inoculum, in contrast to the OBW inoculum, showed a similar relative abundance for all four substrates at the end of the BMP test, thus, indicating a stable methanogenic community (Fig. 5B). In the BREW inoculum, the relative abundance of the Methanomicrobiales population strongly decreased at the end of the BMP test, compared with the initial inoculum, in contrast to an increasing Methanosaetaceae abundance, except for the treatment with the liquid manure substrate (Fig. 5C). In the ENG inoculum, finally, the Methanosaetaceae increased at the end of the BMP test, compared with the inoculum, while the Methanobacteriales decreased in relative abundance, irrespective of the substrate (Fig. 5D). The level of change is, however, less pronounced, compared with the OBW inoculum, as the same dominant populations (i.e. Methanosaetaceae and Methanobacteriales) were maintained.

## Discussion

The BMP of four different organic substrates was determined using four different inocula, originating from full-scale mesophilic anaerobic digesters. Significant differences were detected for the BMP result of the liquid manure and A-sludge substrate. Both substrates also showed indigenous methane production, in contrast to the molasses and bio-refinery waste substrates. However, the resulting methane yield from indigenous methanogenic activity was much lower than in the presence of an inoculum. A clear difference in methanogenic community composition and abundance was observed between the different inocula.

### Inoculum selection influences the BMP result

The determination of the BMP of four different substrates, each by means of four different inocula showed a significant effect of the inoculum on the BMP result for the liquid manure and A-sludge substrates, whereas no significant differences were detected for the molasses and bio-refinery waste.

The absence of an inoculum effect on the BMP result of molasses relates to other (fed-)batch experiments, showing similar methane yield results and COD removal efficiencies in the order of 70–88% for molasses-based wastewaters, irrespective of the operational conditions and selected inoculum (Boopathy and Tilche, 1991; Satyawali and Balakrishnan, 2008). The BMP results of the bio-refinery waste substrate were, like the molasses, also not influenced by the inoculum, yet methane yield was much lower compared with the molasses. This rather low methane yield and conversion efficiency of the bio-refinery waste most likely relates to the fact that the easily biodegradable components are removed during distillation and subsequent ethanol extraction. Indeed, anaerobic treatment of wine distillery wastewater and vinasses after distillation resulted in similar COD conversion efficiencies of maximum 40–50% (Debazua *et al*., 1991; Pérez *et al*., 1999). The absence of significant effect of the inoculum on the final BMP result of the molasses and bio-refinery waste, although a clear difference in the methane production profile could be observed through time between the different inocula (Fig. 2), most likely relates to the presence of higher amounts of soluble biodegradable components in these substrates, especially in the molasses.

The liquid manure and A-sludge substrates did show a significant difference in methane yield when different inocula were used. The low methane yield for the liquid manure with the MAN inoculum is rather surprising, given the fact that the main substrate for the full-scale plant was animal manure. The fact that the COD conversion efficiency of liquid manure remained < 50% for all inocula is a consequence of the presence of recalcitrant lignocellulosic matter (Angelidaki and Ahring, 2000). The BMP results relate to other studies concerning the digestion of manure in which methane yields of 230–300 ml CH_4_ g^−1^ VS and COD conversion efficiencies between 19.4% and 31.1% were reported (Angelidaki and Ahring, 2000; Moller *et al*., 2007; Carrere *et al*., 2009). The liquid manure also showed high indigenous methanogenic activity, compared with the other substrates (Fig. 3). However, methane yield in the absence of an inoculum was much lower than when an inoculum was used, thus, demonstrating the necessity to use an inoculum for BMP testing. The BMP of the A-sludge was also influence by the inoculum. The BMP result of the A-sludge greatly depends on the sludge retention time (SRT) in the A-stage, with a decreasing anaerobic biodegradability at higher SRT values (Ge *et al*., 2013). Our results relate to those reported in earlier studies that showed A-sludge COD conversion efficiencies between 63% and 85%, depending on the SRT in the A-stage, which was 1.8 days in the Breda wastewater treatment plant from which our A-sludge originated (De Vrieze *et al*., 2013a,b; Ge *et al*., 2013).

### The microbial community influences the BMP results

The evaluation of total bacteria and the different methanogenic populations Methanobacteriales, Methanomicrobiales, Methanosaetaceae and Methanosarcinaceae revealed a clear difference between the different inocula. The methanogenic population in the OBW and MAN inocula was similar, which is most likely a consequence of animal manure being the main substrate in both full-scale plants. Indeed, especially a dominance of Methanobacteriales or other hydrogenotrophic methanogens is often observed in manure digesters (St-Pierre and Wright, 2013). The presence of Methanosarcinaceae only in the ENG inoculum can be related to its increased level of total VFA, in contrast to the other inocula, and confirms the presence of certain factors in this inoculum negatively influencing methanogenesis (De Vrieze *et al*., 2012). The high abundance of total methanogens in the BREW inoculum, compared with the other inocula, can be explained by its full-scale plant of origin, which was a UASB reactor. Active retention of microbial biomass in UASB systems allows higher abundances of the slow growing methanogens, and, thus, a higher methanogens : bacteria ratio and absolute concentration of methanogens (Diaz *et al*., 2003; Satoh *et al*., 2007).

Methanogens were only observed in the liquid manure and A-sludge substrates. This relates to the far more favourable circumstances in the liquid manure and A-sludge for methanogenic growth (Table 2), and the fact that both substrates contain animal manure and human faeces respectively. This was confirmed by the indigenous methanogenic activity in the liquid manure and A-sludge. The high abundance of Methanosaetaceae and Methanobacteriales in the liquid manure and A-sludge substrates is in correlation to the OBW and MAN inocula, thus, indicating that both populations play an important role in methane formation from manure.

The high degree of change in the OBW inoculum, irrespective of the substrate, indicates that the methanogenic community in the inoculum had not reached steady state yet, since, in general, during stable anaerobic digestion a static methanogenic community is observed, which is not strongly influenced by a change in substrate (Shin *et al*., 2010; Ritari *et al*., 2012; De Vrieze *et al*., 2013a,b; Regueiro *et al*., 2014; Town *et al*., 2014; Venkatakrishnan *et al*., 2014). The endurance of the Methanomicrobiales population in the treatment with the liquid manure substrate indicates that both the acetoclastic and hydrogenotrophic pathway are used for methane production from liquid manure. This relates to the dominance of hydrogenotrophic methanogenesis, whether or not coupled to syntrophic acetate oxidation, observed during manure digestion, as indicated earlier (Borowski and Weatherley, 2013; St-Pierre and Wright, 2013). Methanosarcinaceae, only present in the ENG inoculum, apparently do not contribute to methane production during the BMP test, as they were not detected in any of the treatments at the end of the BMP-test.

### Methanogenesis might be inhibited in certain inocula

Conductivity, pH and TAN were lower in the BREW inoculum compared with the other inocula, but were typical for UASB type reactors treating liquid waste streams (Leitao *et al*., 2005). The higher pH, conductivity and TAN values in the OBW and MAN inocula samples can be attributed to the treatment of high amounts of manure in both plants, which was not the case in the ENG plant, as previously reported (Angelidaki and Ahring, 1993; Hansen *et al*., 1998; Astals *et al*., 2012). These high pH and TAN values, however, lead to free ammonium concentrations that might be inhibiting methanogenesis. Indeed, the MAN inoculum had a free ammonia concentration of 859 mg N l^−1^ (Table 1), which can be considered well above the minimum inhibitory level for methanogenesis (Angelidaki and Ahring, 1993; Chen *et al*., 2008). This may explain the rather slow start-up of methane production during the BMP tests with the MAN inoculum, and the fact that this inoculum showed the lowest BMP results for molasses, liquid manure and A-sludge substrates, although differences were not always significant, compared with the other inocula (Figs 1 and 2B). Nonetheless, the low VFA concentrations in the inoculum indicate adaptation and increased tolerance of the microbial community to high levels of free ammonia, as well as to the high conductivity in both the OBW and MAN inocula (Chen *et al*., 2008; Schnürer and Nordberg, 2008; De Vrieze *et al*., 2012). Conductivity, pH and TAN were much lower in the ENG inoculum, yet, total VFA reached a value of 1397 mg COD l^−1^, which may point to an unstable microbial community in this inoculum (De Vrieze *et al*., 2012).

### An optimal methanogenic community should be selected

The importance of inoculum selection for BMP testing is very important, as the estimation of an accurate substrate COD conversion efficiency to methane, and therefore price-setting of the substrate, depends upon it. This has led to the development of guidelines to ensure standardized BMP testing (Angelidaki *et al*., 2009). Up until now, however, no guidelines exist concerning the origin and methanogenic community composition or abundance of the BMP inoculum. Nonetheless, in this research, a significant effect of the selected inoculum on the BMP results of two out of four substrates, namely liquid manure and A-sludge was observed (Fig. 1), indicating that the effect of the selected inoculum cannot be ignored, as demonstrated earlier (Chamy and Ramos, 2011; Elbeshbishy *et al*., 2012).

The origin of the inoculum determines the stability and, to a certain extent, the metabolic potential of the inoculum. In our case, the high free ammonia concentration in the MAN inoculum and the increased residual VFA concentrations in the ENG inoculum indicate an unstable methanogenic community. This was confirmed by the lower methane yields and slow start-up for the MAN inoculum and the apparent incomplete COD conversion process in the ENG inoculum (Figs 1 and 2). Indeed, for the ENG inoculum, the plateau phase was not reached after 35 days, which was in contrast to the other inocula (Fig. 2D).

Methanogenic community evaluation revealed the most diverse community in the ENG inoculum; yet, this did not relate to the highest methane yields, nor to completion of the COD conversion process over the course of 35 days, as mentioned earlier. The BREW inoculum, which contained intact granules from a full-scale UASB plant, contained the highest absolute abundance of methanogens, which is reflected in the high initial methane production during the first days of the BMP test (Fig. 2C), especially for the molasses and bio-refinery waste substrates. Hence, it appears that a high initial methanogenic abundance is of crucial importance to ensure high COD conversion efficiencies during BMP testing. Based on these results, we recommend the selection of an inoculum with a high abundance of methanogens, preferably originating from an UASB digester. This was in fact also one of the inocula suggested by Angelidaki and colleagues (2009) to be suitable for BMP testing. These inocula also showed high BMP values for the A-sludge and liquid manure, which are two substrates that contain high levels of solids, indicating that hydrolysis was not the rate-limiting step when using the granular BREW inoculum compared with the other inocula, despite the fact that the granules were not disintegrated. However, since in this research no substrates with high solids content and no indigenous microbial community, such as energy crops, were evaluated for their BMP potential, further research will be required to validate this. Moreover, an in-depth evaluation of the bacterial community composition could provide more information concerning the presence/absence of bacterial populations involved in hydrolysis and other stages of the process. As such, a non-granular inoculum with an equally high methanogenic abundance could even be better to determine the BMP of substrate with high solids content.

In conclusion, this research demonstrated the importance of inoculum selection for BMP testing, as a significant effect of the selected inoculum on the BMP result of two out of four substrates was observed. This inoculum effect was mainly attributed to the abundance of methanogens and a potential inhibiting effect of the inoculum itself. Hence, we concluded that an initial high methanogenic abundance is the crucial factor for high COD conversion efficiencies and reliable BMP results.

## Experimental procedures

### Inocula and substrates

Four different anaerobic inocula sludge samples, originating from full-scale mesophilic anaerobic digestion plants, were used to determine the BMP of four different substrates. The inocula were obtained from: (i) a digester treating OBW in which both animal and vegetable waste are treated, (ii) a plant digesting MAN, (iii) a high-rate UASB reactor treating BREW and (iv) a biogas plant treating a diverse range of substrates, mainly ENG. The characteristics of the different inocula are represented in Table 1.

The substrates were selected because of their difference in terms of composition and origin (Table 2). Molasses were collected from AVEVE (the Netherlands). Liquid manure was obtained from a pig farm. Bio-refinery waste was generated from non-genetically modified poplar, which was pre-treated with alkali extrusion, fermented with yeast and distilled to extract the ethanol produced. The A-sludge originated from the A-stage of the ‘Adsorptions-Belebungsverfahren’ or A/B process for municipal wastewater treatment (Boehnke *et al*., 1997), and was collected from the municipal wastewater treatment plant of Nieuwveer (Breda, the Netherlands).

### BMP batch assay

Prior to the BMP test itself, the inocula were incubated at 34 ± 1°C for 5 days to ensure degassing of the inoculum. The BMPs of the different substrates were determined in triplicate in gastight 120 ml serum flasks with a working volume of 80 ml and a headspace of 40 ml, following the recommendations of Angelidaki and colleagues (2009). First, a specific amount of inoculum was added to each flask to obtain a final VS concentration of 20 g VS l^−1^. Second, substrate was added (except for the negative controls) to obtain a substrate to inoculum ratio of 0.5 g COD g^−1^ VS. Third, tap water was added to acquire a total liquid volume of 80 ml in each bottle, irrespective of the selected inoculum or substrate. For each inoculum, negative controls were run in triplicate, containing the selected inoculum at a concentration of 20 g VS l^−1^, to estimate endogenous methane production. After inoculum and substrate addition, the serum flasks were sealed to avoid air intrusion, and maintain anaerobic conditions, and connected to air-tight gas columns by means of an air-tight needle. These gas columns were placed in a water bath containing distilled water at pH < 4.3 to avoid CO_2_ in the biogas from dissolving. The serum flasks were incubated in a linear shaking water bath (Aqua 12 Plus, Novolab, Geraardsbergen, Belgium) at 34 ± 1°C. Volumetric biogas production was evaluated by means of water displacement in the gas columns. Biogas production was measured on daily basis until day 35, when biogas production ceased in all treatments. Biogas volumes and content were reported at standard temperature (273 K) and pressure (101 325 Pa) (STP) conditions. Methane yield was expressed as the volume of methane per gram of substrate VS, and COD yield as the fraction of substrate COD converted to methane, by means of the ideal gas law.

### Evaluation of the indigenous methanogenic activity of the different substrates

A methanogenic activity test, similar to the BMP test, was implemented to estimate indigenous methanogenic activity of the different substrates in gastight 120 ml serum flasks with a working volume of 60 ml and a headspace of 60 ml. To each flask 60 ml of the substrate was added, after which the flask was sealed to avoid air intrusion and to maintain anaerobic conditions, and incubated in a climate room at 34 ± 1°C for 24 days. Each substrate was evaluated in triplicate. Biogas production was determined using a gas pressure UMS-Tensiometer (Infield 7) device (UMS, München, Germany), and reported at STP conditions. Biogas production and composition were measured after 1, 2, 3, 6, 8, 10, 13, 17, 21 and 24 days.

### Microbial community analysis

Total DNA was extracted from the sludge samples following the protocol of Vilchez-Vargas and colleagues (2013). The quality and quantity of the DNA extracts were analysed on 1% agarose gels and spectrophotometrically, using a Nanodrop ND-1000 Spectrophotometer (Isogen Life Science, IJsselstein, the Netherlands), and 100-fold dilutions of the DNA extracts were prepared to reach a final DNA concentration between 1 and 10 ng μl^−1^.

Real-time PCR (qPCR) was performed on a StepOnePlus Real-Time PCR System (Applied Biosystems, Carlsbad, CA, USA) on triplicate DNA extracts. A reaction mixture of 15 μl was prepared by means of the GoTaq qPCR Master Mix (Promega, Madison, WI, USA), and contained 10 μl of GoTaq® PCR Master Mix, 3.5 μl of nuclease-free water, and 0.75 μl of each primer (final concentration of 375 nM). To this mixture 5 μl of template DNA was added. The qPCR program was performed in a two-step thermal cycling procedure that consisted of a predenaturation step of 10 min at 94°C, followed by 40 cycles of 15 s at 94°C and 1 min at 60°C for total bacteria, using the general bacterial primers P338F and P518r, as described by Ovreas and colleagues (1997). The qPCR program for the methanogenic order Methanobacteriales and the families Methanosaetaceae and Methanosarcinaceae consisted of a predenaturation step of 10 min at 94°C, followed by 40 cycles of 10 s at 94°C and 1 min at 60°C. For quantification of the Methanomicrobiales order an annealing temperature of 63°C was used. The primers for the methanogenic orders Methanomicrobiales and Methanobacteriales, and the families Methanosaetaceae and Methanosarcinaceae were described by Yu and colleagues (2005). The qPCR data were represented as copies per gram of wet sludge. Real-time PCR quality was evaluated by means of the different parameters obtained during analysis with the StepOnePlus software V2.3 (Table S1). PCR product length was verified on a 1% agarose gel.

### Analytical methods

Total solids (TS), VS, TAN, TKN, and total COD were determined according to Standard Methods (Greenberg *et al*., 1992). Soluble COD was measured by means of Nanocolor test kits (Macherey-Nagel, Düren, Germany), following the manufacturer’s instructions. Total P analysis was carried out by means of Jenway 6400 spectrophotometer (Keison Products, Essex, UK). Free ammonia concentration was calculated based on the TAN concentration, pH and temperature. Biogas composition and VFA were determined as described in the SI (S3). The pH was measured with a C532 pH meter (Consort, Turnhout, Belgium), and conductivity (EC) was determined using a C833 conductivity meter (Consort, Turnhout, Belgium).

### Statistical analysis

Statistical analysis of the BMP test results was carried out by means of the SPSS Statistics 22 software (IBM Corp., Belgium). Normality and homoscedasticity (homogeneity of variances) were confirmed using the Kolmogorov-Smirnov test and Levene test, respectively. A one-way analysis of variance test was implemented to evaluate if differences could be observed between the different inocula for each substrate, after which post hoc multiple comparison was carried out by means of the Tukey HSD test at the 5% significance level.

## Supporting Information

**Fig. S1.** Ultimate methane yields of the OBW(), MAN (), BREW () and ENG () inocula. The values are expressed as the volume of methane per gram of VS of the inoculum. Error bars show standard deviations. **Table S1.** Quality control of the parameters for real-time PCR analysis. These parameters were obtained during analysis with the StepOnePlus V2.3 software. The detection limit was calculated as copies of the target 16S rRNA gene fragment per gram wet sludge, and was determined taking both dilution and extraction efficiency into account. **S3.** Biogas and volatile fatty acid analysis.
