# Supplementary material for: Inoculum selection influences the biochemical methane potential of agro-industrial substrates
Source: Microb Biotechnol. 2015 Mar 10;8(5):776–86. doi: 10.1111/1751-7915.12268 (PMC4554466; doi:10.1111/1751-7915.12268)
Supplement: Supplementary file 1 [file mbt20008-0776-sd1.docx]

MANUSCRIPT Microbial Biotechnology Version 1

**Supporting Information**

Title: Inoculum selection influences the biochemical methane potential of agro-industrial substrates.

**Jo De Vrieze^1^, Linde Raport^1,2^, Bernard Willems^2^, Silke Verbrugge^1^, Eveline Volcke^3^, Erik Meers^4^, Largus T. Angenent^5^, Nico Boon^1🖂^**

^1^Laboratory of Microbial Ecology and Technology (LabMET), Ghent University, Coupure Links 653, B-9000 Gent, Belgium

^2^ Innolab, Derbystraat 223, 9051 Sint-Denijs-Westrem, Belgium

^3^Department of Biosystems Engineering, Ghent University, Coupure Links 653, B-9000 Gent, Belgium

^4^Laboratory of Analytical Chemistry and Applied Biochemistry, Ghent University, Coupure Links 653, B-9000 Gent, Belgium

^5^Department of Biological and Environmental Engineering, Cornell University, Ithaca, NY 14853, USA

^🖂^ Correspondence to: Nico Boon, Ghent University; Faculty of Bioscience Engineering; Laboratory of Microbial Ecology and Technology (LabMET); Coupure Links 653; B-9000 Gent, Belgium; phone: +32 (0)9 264 59 76; fax: +32 (0)9 264 62 48; E-mail: [Nico.Boon@UGent.be](mailto:Nico.Boon@UGent.be); Webpage: [www.labmet.Ugent.be](http://www.welcome.to/labMET).

# S1 Methane yield in the negative control treatments


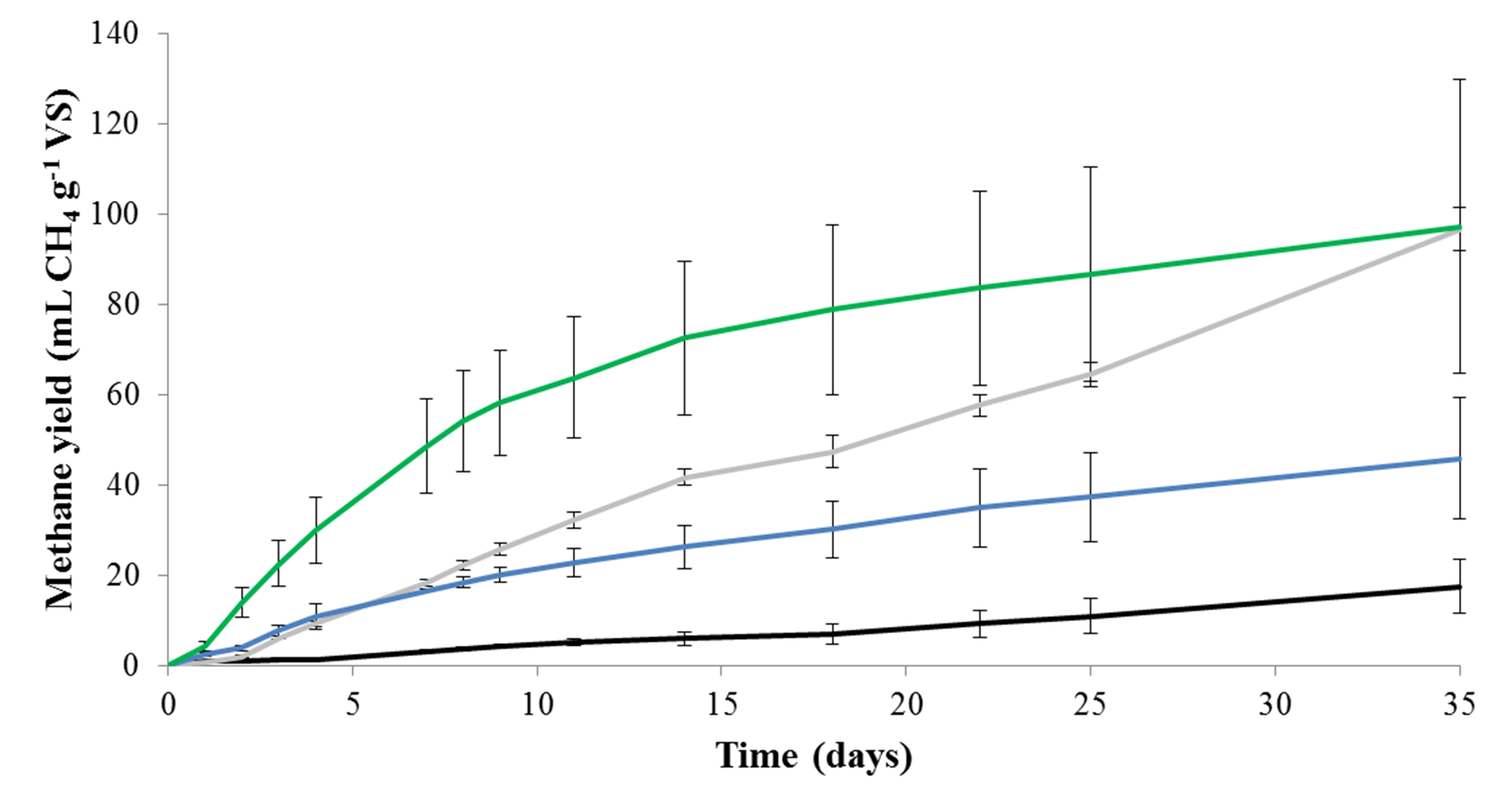


**Figure S1** Ultimate methane yields of the OBW(▬), MAN (▬), BREW (▬), and ENG (▬) inocula. The values are expressed as the volume of methane per gram of VS of the inoculum. Error bars show standard deviations.

# S2 Quality control parameters for real-time PCR analysis

**Table S1** Quality control of the parameters for real-time PCR analysis. These parameters were obtained during analysis with the StepOnePlus V2.3 software. The detection limit was calculated as copies of the target 16S rRNA gene fragment per gram wet sludge, and was determined taking both dilution and extraction efficiency into account.

| Parameter | Slope | R^2^ | Efficiency (%) | Detection limit (copies g^-1^) |
| --- | --- | --- | --- | --- |
| Methanosaetaceae | -3.9 | 1.00 | 80 | 1.53 x 10^4^ |
| Methanosarcinaceae | -3.8 | 1.00 | 83 | 1.45 x 10^4^ |
| Methanobacteriales | -3.4 | 1.00 | 95 | 1.18 x 10^4^ |
| Methanomicrobiales | -3.9 | 1.00 | 82 | 1.08 x 10^4^ |
| Total bacteria | -3.3 | 1.00 | 103 | 9.52 x 10^3^ |

# S3 Biogas and volatile fatty acid analysis

Biogas composition was analysed by means of a Compact GC (Global Analyser Solutions, Breda, The Netherlands), equipped with a Porabond precolumn and a Molsieve SA column. Concentrations of CH_4_ and CO_2_ were determined using a thermal conductivity detector with a lower detection limit of 1 ppmv for each gas component.

The volatile fatty acid (VFA) concentrations were measured using gas chromatography (GC-2014, Shimadzu®, The Netherlands) with a DB-FFAP 123-3232 column (30 m x 0.32 mm x 0.25 µm; Agilent, Belgium) and a flame ionization detector (FID). Liquid samples were conditioned with sulfuric acid and sodium chloride, and 2-methyl hexanoic acid was used as internal standard for quantification of further extraction with diethyl ether. The prepared sample (1 µL) was injected at 200 ºC with a split ratio of 60 and a purge flow of 3 mL min^-1^. The oven temperature increased by 6 ºC min^-1^ from 110 ºC to 165 ºC where it was kept for 2 min. The FID had a temperature of 220 ºC. The carrier gas was nitrogen at a flow rate of 2.49 mL min^-1^.
